# Predicting pathological axillary lymph node status with ultrasound following neoadjuvant therapy for breast cancer

**DOI:** 10.1007/s10549-021-06283-8

**Published:** 2021-06-12

**Authors:** Ida Skarping, Daniel Förnvik, Sophia Zackrisson, Signe Borgquist, Lisa Rydén

**Affiliations:** 1 Division of Oncology and Pathology, Department of Clinical Sciences, Lund University, Lund, Sweden; 2 Department of Clinical Physiology and Nuclear Medicine, Skåne University Hospital, Lund, Sweden; 3 Medical Radiation Physics, Department of Translational Medicine, Skåne University Hospital, Lund University, Malmö, Sweden; 4 Diagnostic Radiology, Department of Translational Medicine, Department of Imaging and Functional Medicine, Skåne University Hospital, Lund University, Lund and Malmö, Sweden; 5 Department of Oncology, Aarhus University Hospital, Aarhus, Denmark; 6 Division of Surgery, Department of Clinical Sciences, Lund University, Lund, Sweden; 7 Department of Surgery, Skåne University Hospital, Lund, Sweden; 8 Aarhus University, Aarhus, Denmark

**Keywords:** Breast cancer, Neoadjuvant chemotherapy, Imaging, Ultrasound, Axillary lymph nodes

## Abstract

**Purpose:**

High-performing imaging and predictive markers are warranted to minimize surgical overtreatment of the axilla in breast cancer (BC) patients receiving neoadjuvant chemotherapy (NACT). Here we have investigated whether axillary ultrasound (AUS) could identify axillary lymph node (ALN) metastasis (ALNM) pre-NACT and post-NACT for BC. The association of tumor, AUS features and mammographic density (MD) with axillary-pathological complete response (axillary-pCR) post-NACT was also assessed.

**Methods:**

The NeoDense-study cohort (*N* = 202, NACT during 2014–2019), constituted a pre-NACT cohort, whereas patients whom had a cytology verified ALNM pre-NACT and an axillary dissection performed (*N* = 114) defined a post-NACT cohort. AUS characteristics were prospectively collected pre- and post-NACT. The diagnostic accuracy of AUS was evaluated and stratified by histological subtype and body mass index (BMI). Predictors of axillary-pCR were analyzed, including MD, using simple and multivariable logistic regression models.

**Results:**

AUS demonstrated superior performance for prediction of ALNM pre-NACT in comparison to post-NACT, as reflected by the positive predictive value (PPV) 0.94 (95% CI 0.89–0.97) and PPV 0.76 (95% CI 0.62–0.87), respectively. We found no difference in AUS performance according to neither BMI nor histological subtype. Independent predictors of axillary-pCR were: premenopausal status, ER-negativity, HER2-overexpression, and high MD.

**Conclusion:**

Baseline AUS could, to a large extent, identify ALNM; however, post-NACT, AUS was insufficient to determine remaining ALNM. Thus, our results support the surgical staging of the axilla post-NACT. Baseline tumor biomarkers and patient characteristics were predictive of axillary-pCR. Larger, multicenter studies are needed to evaluate the performance of AUS post-NACT.

**Supplementary Information:**

The online version contains supplementary material available at 10.1007/s10549-021-06283-8.

## Introduction

Neoadjuvant chemotherapy (NACT) is the recommended treatment option for breast cancer (BC) patients with axillary lymph node (ALN)-positive disease [1]. The accomplishment of pathological complete response (pCR) following NACT, preferably including response in the breast and the axilla, is associated with improved prognosis [2, 3]. To avoid surgical over-treatment of the axilla, that is abstaining from axillary lymph node dissection (ALND) for patients that subsequently are shown to have axillary-pCR, high-performing imaging and/or less invasive surgical staging procedures are needed [4–7]. The SENTINA trial [4] and the Z1071 trial [5] are both prospective multicenter studies. The SENTINA trial was designed to evaluate the timing of sentinel lymph node biopsy (SLNB) in the NACT setting and the objective of the Z1071 trial was to determine the false-negative rate (FNR) for sentinel node (SLN) surgery following chemotherapy in women initially presenting with biopsy-proven cN1 BC. In both studies, the primary endpoint was FNR of SLNB after NACT in patients presenting with upfront cN1 disease. The SENTINA trial and the Z1071 trial showed a FNR of 14% and 13%, respectively, for SLNB performed post-NACT, thus higher than the predefined threshold of 10%. The SLN FNR was not different based on axillary ultrasound (AUS) results; however, using a strategy where only patients with normal AUS undergo SLN surgery reduced the FNR in patients with ≥ two SLNs removed included in the Z1071 trial from 12.6 to 9.8% when preoperative AUS results are considered as part of SLN surgery [8].

AUS is often the first-hand choice for axillary imaging, while more advanced methods for instance magnetic resonance imaging (MRI) and ^18^F-fluorodeoxyglucose positron emission tomography/computed tomography (FDG-PET/CT) are seldom routinely used [9]. At the time point of BC diagnosis, abnormal baseline AUS is routinely followed by ultrasound-guided fine needle aspiration cytology (FNAC); a quick minimally invasive method of axillary staging. FNAC-verified ALNM obviates SLNB, allowing the patient to proceed directly to ALND or, as for the patients in the present study, to NACT followed by ALND [10, 11]. However, current guidelines recommend that SLNB can safely be performed post-NACT for upfront c/pN + patients and used as a discriminator for ALND [12, 13].

From an axillary surgery perspective, correct prediction of axillary-pCR is of utmost interest to enable abstaining from ALND. Identification of predictive imaging biomarkers of axillary-pCR is therefore important. In addition to breast tumor and ALN characteristics, mammographic density (MD) and its association with axillary-pCR, is investigated in this study. The timing of SLNB for BC patients treated with NACT is debated, and current guidelines [11, 12] recommend SLNB performed post-NACT and, in case of benign findings, omission of ALND [4, 5].

In this study, we report results from a well-characterized prospective cohort with AUS performed pre- and post-NACT and detailed pathology data on ALN-metastases (ALNM) from both baseline (pre-NACT) and post-NACT (breast surgery and ALND). In addition, prospectively assessed mammographic density (qualitatively and quantitatively) at the corresponding time points were retrieved. While a large number of studies have investigated the performance of AUS pre-NACT [14, 15], only a few studies have investigated the performance of AUS post-NACT [6, 8, 16–19] and not all report test performance data [8, 17]. We investigated the test performing measures in terms of correctly identifying ALNM of AUS pre-NACT and, most importantly, post-NACT. Since overweight and a lobular BC subtype could be associated with inferior accuracy of AUS [20–22], stratification according to these parameters were performed. We also aimed to investigate the association between AUS parameters, as well as patient and tumor characteristics, respectively, as predictors of axillary-pCR.

## Methods

**Fig. 1:**
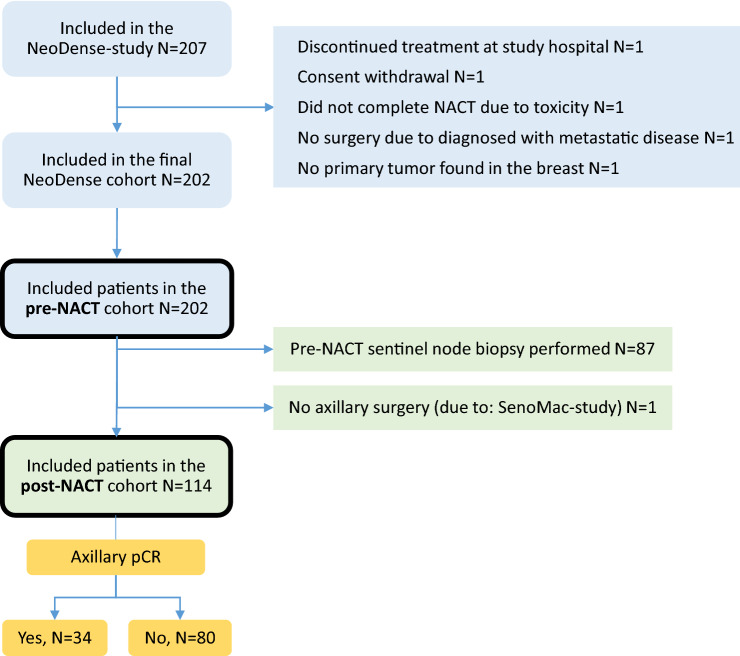
Flowchart

The NeoDense-study cohort, a part of the SCAN-B study [23] (Clinical Trials ID NCT02306096), is a prospective cohort of BC patients receiving NACT during 2014–2019 at two sites within Skåne University Hospital, Sweden as previously described [24]. At diagnosis, patients eligible for NACT were included in the study following written consent (*N* = 207), of whom five patients were excluded due to ineligibility (Fig. 1) [25]. All patients with c/pN + pre-NACT were subject to ALND according to clinical routine and the Swedish National Guidelines at the time of study inclusion (Supplementary Material 1).

### Pre-NACT cohort

For the assessment of AUS at the pre-NACT time point, the whole NeoDense-cohort was used (*N* = 202), in order to include both baseline AUS abnormal and normal assessments. SLNB was performed pre-NACT for patients with clinically and AUS node-negative disease (cN0) (*N* = 87). Hence, the pathological diagnosis of ALN at baseline were based on assessment of SLNs in 87 patients and FNAC in 115 in patients with cytology verified ALNM (Fig. 1). SLNB was performed prior to study inclusion in 78 patients, and for these patients, the axillary evaluation of the preceding diagnostic AUS was used in the statistical analyses of the pre-NACT cohort.

### Post-NACT cohort

From the 202 patients enrolled in the NeoDense study, we report on 114 (56%) who had a cytology verified ALNM at baseline and had an ALND performed post-NACT. At the post-NACT time point, patients having pre-NACT SLNB-performed (*N* = 87) were excluded and *N* = 1 patient was excluded due to being part of the SenoMac-study (Clinical Trials ID NCT02240472) and thus no ALND was performed.

The term “axillary-pCR” commonly used in the literature, meaning no remaining invasive cancer in the axilla following NACT, is in this study only used for patients with FNAC-verified metastases at baseline; the term axillary-pCR was only applicable to patients with no previous surgical removal of ALN (due to SLNB).

### Clinical data

Referring to the pre-NACT cohort (*N* = 202): a total of *N* = 196 (97%) patients received standard chemotherapy regimen [3 × fluorouracil, epirubicin and cyclophosphamide (FEC), or epirubicin and cyclophosphamide (EC) + 3 × docetaxel (or equivalent series of paclitaxel)], or in the reversed order. In the case of human epidermal growth factor receptor 2 (HER2) overexpression BC (*N* = 49), HER2-blockade was added [*N* = 46, whereof 94% received double HER2-blockade (trastuzumab and pertuzumab), and the remaining three patients received single trastuzumab]. Data on clinical and pathological parameters were gathered from study-specific forms, medical charts, and clinical pathology reports.

### Pathology evaluation

FNAC of suspicious ALN was performed pre-NACT according to clinical routine by breast radiologists prior to study inclusion. Standard procedure included aspiration with 22 Gauge needle (0.7 mm × 50.0 mm). All pathological interpretation was performed according to clinical routine at the pathology department by board certified cytopathologists. pCR was defined as no residual invasive cancer foci in the breast and axilla (ypT0/is ypN0), in accordance with current guidelines [26].

### Imaging

Each patient had imaging examinations performed of the breast (mammography and ultrasound and parts of the cohort also breast tomosynthesis) and the axilla (AUS) at three time points: pre-NACT, after two series of NACT (during NACT), and post-NACT; the timing was mirroring clinical routine, and a detailed timeline of the cohort is already published [24]. Ultrasound assessment of ALNs was performed by experienced breast radiologists (specially trained and working at a breast imaging center *N* = 13) and were considered abnormal or normal by evaluating the following criteria: nodal size, cortical thickening, hilar effacement, echogenicity, and shape [27]. No study-specific criteria for abnormal ALN was used; the assessment of normal/abnormal was at the discretion of the evaluating radiologist. Size, shape, cortex thickness above 3–4 mm, hilar effacement, and echogenicity were in a combined overall assessment used in the clinic by the radiologist to discriminate between normal/abnormal nodes. At the time of study inclusion, Breast Imaging-Reporting and Data System (BI-RADS) for AUS categorization was not used. The radiologists prospectively filled in study-specific forms at the time of AUS examinations including number of abnormal ALNs and their size(s) [28, 29] and echogenicity. For the post-NACT cohort, the number of valid long and short-axis measurement, respectively, for the abnormal AUS only at the different time points are presented in Table 2. The details of the ultrasound machines used are presented in Supplementary Material 2. Axillary radiological complete response (rCR) was defined as no abnormal findings (i.e., findings indicating malignancy) by AUS. Mammographic density was assessed both qualitatively by radiologists according to BI-RADS [30], and quantitatively with the automated software Volpara^™^ (version 1.5.4.0, Volpara Solutions Limited, Wellington, New Zealand) [31]. The breast tumor was marked with a radiopaque clip prior to NACT according to clinical routine, while no marking was performed of abnormal ALNs.

### Statistics

We summarized cohort baseline characteristics, including pathology results from the breast and axilla. We calculated descriptive statistics according to axillary-pCR for ultrasound features of the breast tumor and ALN at three time points (baseline, during NACT, and post-NACT).

Test performance: For pre- and post-NACT cohort, we used axillary node-stage by AUS as a test for axillary node-stage by pathology/cytology, both at baseline (*N* = 202) and post-NACT (patients having FNAC-verified ALNM at baseline as well as ALND performed, *N* = 114). We estimated test performance measures; sensitivity, specificity, positive, and negative predictive value (PPV and NPV) with 95% confidence interval (CI) for the AUS-pathology association at baseline and post-NACT. Subgroup analyses were performed according to body constitution and histopathological subtype.

#### Prediction models of axillary-pCR: post-NACT cohort

We furthermore used simple logistic regression to assess whether baseline patient [age, body mass index (BMI), menopausal status], tumor characteristics [estrogen receptor (ER), HER2, Ki67], histopathological subtype, and imaging characteristics (tumor response, MD, and the number of abnormal ALN by AUS) were associated with axillary-pCR (note that absence of ALNM is considered as outcome). In these models, axillary-pCR was the dependent variable, whereas the individual characteristic was included as an independent variable. To establish the independent association of these characteristics and axillary-pCR, we also conducted a fully adjusted multivariable logistic model. The independent variables in this axillary-pCR-model were deduced from simple and multivariable logistic regression models of different AUS parameters of abnormal ALN (number, long-axis, long/short-axis ratio), ultrasound breast tumor parameters (size and response), and MD at three different time points and their association with axillary-pCR. Each multivariable model included the baseline covariates from the previous model: model 1, age; model 2, model 1 + BMI and menopausal status; and, model 3, model 2 + ER-status + HER2-status + Ki67 (all from core biopsies of the breast tumor at baseline).

#### Statistical software

For the test performance measures calculations, MedCalc Statistical Software version 19.2.6 (MedCalc Software Ltd, Ostend, Belgium; https://www.medcalc.org; 2020) was used. Otherwise, IBM SPSS Statistics for Windows, version 26 (IBM Corp., Armonk, N.Y., USA) was used.

## Results

### Descriptive results

The baseline characteristics of the pre- and post-NACT cohort are displayed in Table 1. Invasive ductal carcinoma was the most common histopathological subtype in both the pre- and post-NACT cohort (165 of 202 (82%) and 97 of 114 (85%), respectively), followed by invasive lobular carcinoma (16 of 202 (8%) and 9 of 114 (8%), respectively). Regarding MD, most patients had intermediate MD (BI-RADS b or c combined accounted for 165 of 202 (82%) and 91 of 114 (80%) of the pre- and post-NACT cohort, respectively). The axillary-pCR rate was 30% (34 of 114).

**Table 1:** Pre- and post-NACT cohort: patient, tumor, and axillary characteristics pre-NACT, and pathological/radiological ALN-status post-NACT

	Pre-NACT cohort (*N* = 202)	Post-NACT cohort (*N* = 114)
*Pre-NACT*		
Age at diagnosis, median (IQR)	53 (45—62)	54 (45—63)
BMI, median (IQR)	26 (22—29)	26 (23—29)
Menopausal status, *N* (%)		
Premenopausal	96 (47.5)	51 (44.7)
Postmenopausal	106 (52.5)	63 (55.3)
Tumor size as assessed by mammography, median (IQR)	30 (20—40)	27 (19—35)
No detectable tumor, *N* (%)	11 (5.4)	8 (7.0)
Tumor size not assessable, *N* (%)	10 (5.0)	7 (6.1)
Test not performed, *N* (%)	1 (0.5)	0 (0)
Tumor size as assessed by ultrasound, median (IQR)	28 (19—35)	24 (17—34)
No detectable tumor, *N* (%)	2 (1.0)	1 (0.9)
Tumorsize not assessable, *N* (%)	5 (2.5)	2 (1.8)
Estrogen receptor status, *N* (%)		
Positive (≥ 10%)	121 (59.9)	83 (72.8)
Negative (< 10%)	81 (40.1)	31 (27.2)
Progesterone receptor status, *N* (%)		
Positive (≥ 10%)	103 (51.0)	70 (61.4)
Negative (< 10%)	98 (48.5)	43 (37.7
Missing	1 (0.5)	1 (0.9)
HER2 receptor status^a^, *N* (%)		
Positive	49 (24.3)	24 (21.1)
Negative	153 (75.7)	90 (78.9)
Ki67^b^, *N* (%)		
Low	11 (5.4)	8 7.0)
Intermediate	30 (14.9)	20 (17.5)
High	159 (78.7)	85 (74.6)
Missing	2 (1.0)	1 (0.9
Histopathological subtype, *N* (%)		
Ductal	165 (81.7)	97 (85.1)
Lobular	16 (7.9)	9 (7.9)
Other	12 (5.9)	4 (3.5)
Missing	9 (4.5)	4 (3.5)
Mammographic density		
VBD%, median (IQR)	11.5 (7.6–18.3)	10.4 (6.9–17.2)
Missing, *N* (%)	9 (4.5)	4 (3.5)
BI-RADS, N (%)		
A	9 (4.5)	6 (5.3)
B	74 (36.6)	43 (37.7)
C	91 (45.0)	48 (42.1)
D	27 (13.4)	17 (14.9)
Missing	1 (0.5)	1 (0.5)
Abnormal ALN by AUS, *N* (%)		
Yes	123 (60.9)	109 (95.6)
No	79 (39.1)	5 (4.4)
*Post-NACT*		
Axillary-pCR, *N* (%)		
Yes	N/A	34 (29.8)
No	N/A	80 (70.2)
Abnormal ALN by AUS, *N* (%)		
Yes	41 (20.3)	38 (33.3)
No	155 (76.7)	75 (65.8)
Missing	6 (3.0)	1 (0.9)

*BMI* body mass index, *HER2* human epidermal growth factor receptor 2, *VBD%* volumetric breast density percentage, *BI-RADS* Breast Imaging-Reporting and Data System, *ALN* axillary lymph nodes, *AUS* axillary ultrasound, *pCR* pathological complete response

^a^If the tumor was assessed as 3 + with immunohistochemistry and/or amplified with in situ hybridization

^b^Tumors were considered as low, intermediate, or highly proliferative according to laboratory specific cutoffs (site 1: low 0–20%; intermediate 21–30%; high 31–100%, site 2: low 0–14%; intermediate 15–24%; high 25–100%) for proportion of cells staining positive for Ki67

The number and proportion of abnormal ALNs by AUS decreased during NACT in both the axillary-pCR and non-axillary-pCR group (Table 2). Post-NACT, the proportion of normal ALN-status by AUS was 77% (26 of 34) in the axillary-pCR group and 61% (49 of 80) in non-axillary-pCR group. Of the 78 patients with SLNB prior to inclusion, 83% (*N* = 65) had no abnormal findings by AUS.

**Table 2:** Post-NACT cohort: ultrasound features of breast tumor and ALN, at baseline, during NACT, and post-NACT according to axillary-pCR

		Baseline			During NACT (after 2 cycles)			Post-NACT		
		*N* = 114	Axillary-pCR *N* = 34	Non axillary-pCR *N* = 80	*N* = 114	Axillary-pCR *N* = 34	Non axillary-pCR *N* = 80	*N* = 114	Axillary-pCR *N* = 34	Non axillary-pCR *N* = 80
US breast diameter	Tumor size (breast) mm, median (IQR)	24 (17–35)	22 (17–33)	25 (17–35)	16 (11–25)	14 (7–24)	18 (12–26)	9 (0–15)	6 (0–12)	10 (6–15)
	Missing, *N* (%)	2 (1.8%)	1 (2.9%)	1 (1.3%)	5 (4.4%)	1 (2.9%)	4 (5.0%)	3 (2.6%)	1 (2.9%)	2 (2.5%)
AUS assessment of all ALN	Number of abnormal ALN by AUS, mean (95% CI)	1.80 (1.62–1.98)	1.91 (1.58–2.25)	1.75 (1.54–1.96))	1.26 (1.06–1.46)	1.09 (0.74–1.44)	1.33 (1.08–1.58)	0.55 (0.38–0.71)	0.35 (0.11–0.59)	0.63 (0.42–0.85)
	Missing, *N* (%)	0	0	0	1 (0.9%)	0	1 (1.3%)	1 (0.9%)	0	1 (1,3%)
	Abnormal ALN by AUS									
	Yes, *N* (%)	109 (95.6%)	33 (97.1%)	76 (95.0%)	81 (71.0%)	23 (67.6%)	58 (72.4%)	38 (33.3%)	8 (23.6%)	30 (37.4%)
	No, *N* (%)	5 (4.4%)	1 (4.4%)	4 (5.0%)	32 (28.1%)	11 (32.4%)	21 (26.3%)	75 (65.8%)	26 (76.5%)	49 (61.3%)
	Missing, *N* (%)	0	0	0	1 (0.9%)	0	1 (1,3%)	1 (0.9%)	0	1 (1.3%)
	Negative radiological ALN-status	4.4%	4.4%	5.0%	28.3%	32.4%	26.6%	66.3%	76.5%	62.0%
AUS diameter in abnormal ALN	Longest diameter of pathological ALN, mm median (IQR)	18 (13–23)	17.5 (12–27.75)	18.5 (13.75–23)	14 (11–20)	13 (10–20)	14 (12–19	13 (9.25–16)	16 (12.75–20.25)	12.5 (9–15)
	Valid, *N* (%)	102 (93.6%)	32 (97.0%)	70 (92.1%)	75 (92.6%)	23 (100%)	52(89.7%)	38 (100%)	8 (100%)	30 (100%)
	Shortest diameter of pathological ALN, mm median (IQR)	10 (7–12)	10 (7–16.5)	10 (7–12)	7 (5–10)	5 (4–7.75)	8.5 (5.75–10.25)	6 (4–7.25)	6 (4–9)	6 (4–7)
	Valid, *N* (%)	81 (74.3%)	25 (75.8%)	56 (73.4%)	60 (74.0%)	18 (78.3%)	42 (72.4%)	30 (78.9%)	7 (87.5%)	23(76.7%)
	Ratio of long/short diameter (median, IQR)	1.77 (1.34–2.39)	1.47 (1.20–2.55)	1.91 (1.39–2.39)	2.06 (1.47–2.54)	2.35 (1.42–3.78	2.06 (1.47–2.43)	2.07 (1.71–3.04)	2.78 (1.60- 4.29)	2.07 (1.71–2.85)
	Valid, *N* (%)	79 (72.4%)	25 (75.8%)	54 (71.1%)	57 (70.4%)	18 (78.3%)	39 (67.2%)	30 (78.9%)	7 (87.5%)	23 (76.7%)

*ALN* axillary lymph nodes, *NACT* neoadjuvant chemotherapy, *pCR* pathological complete response, *US* ultasound, *AUS* axillary ultrasound

### Test performance

**Fig. 2:**
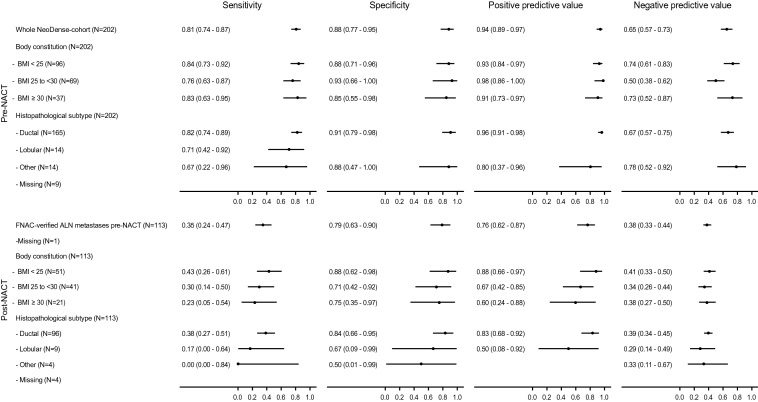
Test performance measures of AUS pre- and post-NACT. Stratification according to body constitution and histopathological subtype

Test performance measures of AUS pre- and post-NACT were stratified according to BMI and histological subtype, are presented in Fig. 2. Pre-NACT, a total of 123 of 202 (61%) met abnormal AUS criteria (according to the expertise judgment by the radiologist), the corresponding number post-NACT was 38 of 114 (33%). AUS showed better performance in terms of identifying ALNM pre-NACT (*N* = 202) as reflected by the PPV of 0.94 (95% CI 0.89–0.97) and sensitivity of 0.81 (95% CI 0.74–0.87). The performance of AUS was inferior post-NACT (*N* = 114); PPV 0.76 (95% CI 0.62–0.87) and sensitivity 0.35 (95% CI 0.24–0.47). Stratified analyses according to BMI and histological subtype pre- and post-NACT showed no differences (Fig. 2).

### Prediction models of axillary-pCR: post-NACT cohort

Baseline characteristics positively associated with accomplishing axillary-pCR in the simple and multivariable logistic regression analysis (*N* = 114) were: premenopausal status (OR 0.08 95%CI 0.01–0.82), ER-negativity (OR 9.05 95%CI 2.09–39.14), HER2-overexpression (OR 6.18 95%CI 1.62–23.56), and mammographic dense breasts (OR 6.98 95%CI 1.54–31.62) (Table 3). Tumor response as assessed with ultrasound (decrease ≥ 30% in largest diameter) between baseline and “during NACT” showed association with axillary-pCR in the unadjusted model (OR 2.60 95%CI 1.11–6.07); however, this association was not retained in the multivariable model (OR 1.48 95%CI 0.43–5.08). The fully adjusted multivariable model including the 114 patients (adjusting for age, BMI, menopausal status, ER, HER2, and Ki67) is displayed in Supplementary Material 3, showing that the odds ratio for accomplishing axillary-pCR increased with the decreasing number of abnormal ALNs on AUS during (OR 0.46 95%CI 0.25–0.83) and post-NACT (OR 0.58 95%CI 0.30–1.10) (Supplementary Material 3).

**Table 3:** Post-NACT cohort: simple and multivariable logistic regression analysis of baseline tumor, patients characteristics, and imaging characteristics during/post-NACT as predictors of axillary-pCR following NACT

Variables	Simple logistic regression			Multivariable* logistic regression		
	OR (95% CI)	*p* value	*N*	OR (95% CI)	*p* value	*N*
Patient and tumor characteristics (baseline)						
Age (continuous)	0.97 (0.94–1.01)	0.11	114	0.92 (0.84–1.01)	0.08	94
BMI (continuous)	0.97 (0.89–1.07)	0.55	114	0.94 (0.82–1.09)	0.42	94
Postmenopausal	1.29 (0.58–2.87)	0.54	114	0.08 (0.01–0.82)	0.03	94
ER-negativity	6.53 (2.65–16.07)	< 0.01	114	9.05 (2.09–39.14)	< 0.01	94
HER2-overexpression	5.53 (2.14–14.25)	< 0.01	114	6.18 (1.62–23.56)	< 0.01	94
Ki67 (continuous)	1.03 (1.01–1.05)	< 0.01	113	1.02 (0.99–1.05)	0.31	94
Histopathological subtype—ductal (ref)			97			86
Lobular	0.64 (0.13–3.26)	0.59	9	0.72 (0.05–10.34)	0.81	6
Other	0.74 (0.07–7.45)	0.80	4	4.03 (0.17–97.16)	0.39	2
Imaging characteristics						
Tumor response during NACT (decrease ≥ 30%, yes/no)	2.60 (1.11–6.07)	0.03	107	1.48 (0.43–5.08)	0.53	94
Mammographic density: BI-RADS dichotomized post-NACT (ref A/B”non-dense”)			48			42
C/D (”dense”)	2.85 (1.16–6.97)	0.02	58	6.98 (1.54–31.62)	0.01	52
Number of abnormal ALN post-NACT by AUS	0.66 (0.39–1.13)	0.13	113	0.58 (0.28–1.24)	0.16	94

*NACT* neoadjuvant chemotherapy, *pCR* pathological complete response, *OR* odds ratio, *CI* confidence interval, *BMI* body mass index, *ER* estrogen receptor, *HER2* human epidermal growth factor receptor 2, *BI-RADS* Breast Imaging-Reporting and Data System, *ALN* axillary lymph nodes, *AUS* axillary ultrasound

^*^adjusted for: age, BMI, menopausal status, ER, HER2, Ki67, histopathological subtype, number of positive ALN by ultrasound post-NACT, tumor size decrease (by ultrasound) ≥ 30% during NACT, and mammographic density (BI-RADS dichotomized post NACT)

## Discussion

For BC patients receiving NACT, reliable imaging is needed both at baseline, in the initial staging-situation for well-grounded systemic treatment decisions, as well as post-NACT to optimize surgical treatment decisions. It is important to evaluate the performance of AUS, as has been studied multiple times before at the initial staging (pre-NACT or pre-primary BC surgery) [14, 15], but reported in a few previous studies post-NACT [6, 16, 18, 19]. We present results of a well-characterized prospective cohort with extensive pathology data (complete data cytology proven ALNM at baseline and ALND post-NACT) and a detailed study protocol with sequential imaging (pre, during, and post-NACT). Adding information to previously published studies, we present clinically valuable performance measures of AUS post-NACT. In addition to the many studies presenting nomograms (predominantly baseline data) for prediction of axillary-pCR [17, 32–38], this study presents novel findings of the association between MD and axillary-pCR.

### Test performance

#### AUS pre-NACT

Our results show that baseline AUS could, to a large extent, correctly identify ALNM; the sensitivity, specificity, and PPV were satisfactory. However, a NPV of 0.65 (95% CI 0.57–0.73) shows that pre-NACT AUS has limitations to correctly identify metastasis of any size in the axilla. This finding supports current guidelines that patients with clinically and AUS normal axilla at baseline can be staged by SLNB post-NACT without missing important information. The literature shows diverse sensitivity and specificity for the diagnosis of ALNM at baseline with AUS, ranging from 49 to 87% and 53 to 97%, respectively [14, 15]. This variety might partly be explained by the lacking consensus for imaging characteristics or scoring systems for abnormal ALN by AUS [15] and that ultrasound is a modality that has high intra- and inter-observer variability [39].

#### AUS post-NACT

In more recent years, classification systems of ultrasound evaluation of ALN post-NACT have been presented to determine important ALN characteristics to consider post-NACT [40]. Importantly, in our study, the sensitivity of AUS post-NACT (identifying ALNM) was considerably lower in comparison to pre-NACT, but the specificity and PPV were acceptable. Thus, AUS could not identify all (subsequent pathology-verified) ALNM. Previous studies have shown sensitivity and specificity rates for AUS (of identification of ALNM) post-NACT of 50–60% and 60–77%, respectively [6, 18]. In the SN FNAC study [19], the PPV and NPV of AUS post-NACT were slightly higher than in the present study (PPV 81% and NPV 48% in SN FNAC-study in comparison to PPV 76% and NPV 38% in our study). These results are to be expected since ultrasound is reflecting a macroscopic feature in contrast to the remaining microscopic findings in the pathology specimen. The study samples are similar (N ranging from 139–157 [6, 18, 19]) to ours except for the large cohort in the SENTINA trial [16].

#### Other modalities

Several strategies in improving the diagnostic performance of axillary staging by imaging have been proposed. In our study, we used a limited number of easily accessible, clinically established AUS parameters [41]. Also, other imaging modalities for axillary staging pre- and post-NACT must be mentioned. In the primary staging-setting, a review by Marino et al*.* showed pooled estimates of the sensitivity of 75–80% and 59–69% for MRI and FDG-PET/CT, respectively, and the corresponding estimates for specificity were 89–91% and 90–95% for MRI and FDG-PET/CT, respectively [15]. When examining the axilla post-NACT, a study using MRI (*N* = 65) has shown a PPV of 67% and a NPV of 66% of biopsy-proven ALNM pre-NACT in terms of predicting axillary-pCR [42]; the corresponding numbers for AUS in our study was PPV 38% and NPV 76%. A comparative study between AUS, MRI, and FDG-PET/CT presented post-NACT sensitivity of axillary imaging in detecting ALNM to be 70% for AUS (*N* = 106), 61% for MRI (*N* = 88), and 63% for FDG-PET/CT (*N* = 32) [43]. Another study comparing different modalities’ [ultrasound (*N* = 135), MRI (*N* = 136), and FDG-PET/CT (*N* = 99)], and combinations thereof, test performance measures post-NACT, showed NPV ranging from 28 to 48%, the latter from the combination of AUS and MRI [6]. In conclusion, studies of these advanced imaging modalities post-NACT have a limited number of study participants and a comprehensive overview of axillary imaging post-NACT, including AUS, is thus warranted.

#### AUS, BMI, and histopathological subtype

Since clinical axillary palpation might be more challenging in overweight/obese patients, AUS is of even greater importance for these patients. In overweight and obese patients AUS could be afflicted with inferior performance due to technical challenges and obesity-related ALN-alterations [44, 45]; however, studies of baseline AUS points toward no impediment [45]. To the best of our knowledge, not previously reported in the literature, we present results of test performance of AUS post-NACT in relation to BMI. In adherence to studies of AUS at baseline, we found no difference in AUS performance according to BMI at either time point. Previous studies [20, 21] have indicated inferior accuracy of AUS in lobular cancer. In the present study, we found inconclusive results in terms of AUS performance at each time points due to insufficient number of patients in the lobular histopathology groups (*N* = 14 pre-NACT and *N* = 9 post-NACT).

### Prediction models of axillary-pCR: post-NACT cohort

An important finding in our study is the results from our simple logistic regression analysis of patient and tumor characteristics: the associations between ER-negativity and HER2-overexpression, respectively, and axillary-pCR were more pronounced than for many of the imaging characteristics of the breast and the axilla. Our results are in line with previous studies presenting predictive models, recognizing the importance of pre-NACT tumor characteristics [17, 32–38, 46]. These studies have similar odds ratio of axillary-pCR as in the present study, thus adding credibility to our results. Similar to the tumor in the breast, ALN response to NACT is dependent on BC tumor subtype [47].

### Mammographic density

Mammographic density, reflecting the radiodense stroma and epithelium of the breast on a mammogram [48], is associated with increased risk of BC development [49], higher risk of recurrence [50], and possibly poorer response to treatment [51, 52], although inconsistent results have been presented [24]. BC tumors in mammographic dense breasts are often larger at diagnosis and have positive ALN [53], thus justifying exploring the association between MD and rate of axillary-pCR. We did not find any association between MD assessed with Volpara^™^ and the likelihood of accomplishing axillary-PCR. In contrast, the BI-RADS assessment showed that dense breasts (BI-RADS c/d) were associated with higher odds ratio of accomplishing axillary-pCR in comparison to non-dense (BI-RADS a/b), an association more pronounced at the later time points. To the best of the authors’ knowledge, no previous studies have addressed MD *vs.* axillary-pCR.

### Future perspective

The timing of SLNB is under scientific and clinical debate [4]. In 40–65% of patients with positive SLN at baseline, the SLN(s) is expected to be the only positive ALN, meaning that many of these patients do not have ALNM left in the axilla during and post-NACT [54, 55]. SLNB offers a reliable staging at baseline [56]; in patients with a benign SLNB pre-NACT, it is considered safe to omit further axillary surgery, conditionally not progressing during NACT [57]. Patients in our cohort were treated according to this clinical algorithm. Correspondingly, there is an ongoing discussion of alternative treatment strategies to ALND for upfront ALN-positive patients with axillary-rCR post-NACT. To reduce the morbidity related to ALND [58], less invasive procedures and treatment strategies are warranted. High demands are put on imaging, of which ultrasound is considered to be the preferred choice for axillary assessment [9], and used as a discriminator for patients eligible for SLNB [9]. Likewise, performed either pre-NACT, or as current guidelines recommend, post-NACT [12, 13]; the test performance measures of SNLB must be high-level, most importantly with low FNR [59]. Our results indicate that AUS is a good predictor of ALNM at baseline, supporting abstaining from SLNB before NACT. However, AUS assessment post-NACT was not able to correctly diagnose remaining tumor deposits in patients with ALNM at the time of diagnosis.

### Strengths and limitations

Our study has many strengths, including the prospective design with detailed data on patient, tumor, and breast characteristics at several time points. Since the guidelines are currently changing, recommending SLNB post-NACT, a similar study to our study and the SENTINA study [4] will be difficult to perform in the future. Possibly, access to breast MRI, could have been beneficial; however, axillary-MRI (provided a dedicated axillary protocol) has a minor role in axillary assessment [15]. Applicable to many imaging studies of the axilla, the lack of a standardized system of reporting findings (e.g., BI-RADS [30]) contributes to a wide variety of AUS test performance measures. To the best of the authors´ knowledge, no guidelines exist on a national or European level regarding assessment of abnormal/normal ALN. Another shortcoming is the incomplete data on ALN short-axis measures (used in the RECIST criteria [60]). Consequently, only a limited number of patients had data on long/short axis ratio, an established measure mirroring the shape of the ALN (round or elongated) [41]. At baseline, according to clinical routine, FNAC was used to verify abnormal ALN by AUS. However, core biopsy to the ALN is considered to have higher diagnostic accuracy and is currently introduced [61]. Selection bias should be briefly addressed; although many patients had a positive ALN at baseline due to NACT being a preferred treatment option for patients with cytology/pathology-verified ALN at baseline, an abnormal ALN-status by AUS was not an inclusion criterion, and the selection bias should thus be minor.

## Conclusion

Prior to NACT, AUS could, to a large extent, correctly identify abnormal ALN, supporting the omission of SLNB pre-NACT. In contrast, AUS alone is not sufficient to determine remaining ALNM post-NACT, whereas tumor biomarkers at baseline are predictive of axillary-pCR. We found no difference in AUS performance according to BMI at any time point. Larger multi-center studies are needed to evaluate the performance of AUS post-NACT. Investigation of other imaging modalities for treatment evaluation post-NACT is encouraged.

## Supplementary Information

Below is the link to the electronic supplementary material.

Supplementary file1 Study time line and treatment algorithm (PPTX 4476 kb)

Supplementary file2 (DOCX 15 kb)

Supplementary file3 (DOCX 30 kb)

## Data Availability

The datasets used and/or analyzed during the current study are available from the corresponding author on reasonable request.

## Abbreviations

NACT: Neoadjuvant chemotherapy
BC: Breast cancer
ALN: Axillary lymph nodes
pCR: Pathological complete response
ALND: Axillary lymph node dissection
SLNB: Sentinel lymph node biopsy
FNR: False-negative rate
SLN: Sentinel node
AUS: Axillary ultrasound
MRI: Magnetic resonance imaging
FDG-PET/CT: ^18^F-fluorodeoxyglucose positron emission tomography/computed tomography
FNAC: Fine needle aspiration cytology
ALNM: Axillary lymph nodes metastases
HER2: Human epidermal growth factor receptor 2
BI-RADS: Breast Imaging-Reporting and Data System
rCR: Radiological complete response
PPV: Positive predictive value
NPV: Negative predictive value
CI: Confidence interval
BMI: Body mass index
ER: Estrogen receptor

## References

[1] Burstein HJ, Curigliano G, Loibl S, Dubsky P, Gnant M, Poortmans P, Colleoni M, Denkert C, Piccart-Gebhart M, Regan M (2019). Estimating the benefits of therapy for early-stage breast cancer: the St. Gallen International Consensus Guidelines for the primary therapy of early breast cancer 2019. Ann Oncol.

[2] von Minckwitz G, Untch M, Blohmer JU, Costa SD, Eidtmann H, Fasching PA, Gerber B, Eiermann W, Hilfrich J, Huober J (2012). Definition and impact of pathologic complete response on prognosis after neoadjuvant chemotherapy in various intrinsic breast cancer subtypes. J Clin Oncol.

[3] Cortazar P, Zhang L, Untch M, Mehta K, Costantino JP, Wolmark N, Bonnefoi H, Cameron D, Gianni L, Valagussa P (2014). Pathological complete response and long-term clinical benefit in breast cancer: the CTNeoBC pooled analysis. Lancet.

[4] Kuehn T, Bauerfeind I, Fehm T, Fleige B, Hausschild M, Helms G, Lebeau A, Liedtke C, von Minckwitz G, Nekljudova V (2013). Sentinel-lymph-node biopsy in patients with breast cancer before and after neoadjuvant chemotherapy (SENTINA): a prospective, multicentre cohort study. Lancet Oncol.

[5] Boughey JC, Suman VJ, Mittendorf EA, Ahrendt GM, Wilke LG, Taback B, Leitch AM, Kuerer HM, Bowling M, Flippo-Morton TS (2013). Sentinel lymph node surgery after neoadjuvant chemotherapy in patients with node-positive breast cancer: the ACOSOG Z1071 (Alliance) clinical trial. JAMA.

[6] You S, Kang DK, Jung YS, An YS, Jeon GS, Kim TH (2015). Evaluation of lymph node status after neoadjuvant chemotherapy in breast cancer patients: comparison of diagnostic performance of ultrasound, MRI and (1)(8)F-FDG PET/CT. Br J Radiol.

[7] Simons JM, van Nijnatten TJA, van der Pol CC, Luiten EJT, Koppert LB, Smidt ML (2019). Diagnostic accuracy of different surgical procedures for axillary staging after neoadjuvant systemic therapy in node-positive breast cancer: a systematic review and meta-analysis. Ann Surg.

[8] Boughey JC, Ballman KV, Hunt KK, McCall LM, Mittendorf EA, Ahrendt GM, Wilke LG, Le-Petross HT (2015). Axillary ultrasound after neoadjuvant chemotherapy and its impact on sentinel lymph node surgery: results from the American College of Surgeons Oncology Group Z1071 Trial (Alliance). J Clin Oncol.

[9] Slanetz PJ, Moy L, Baron P, diFlorio RM, Green ED, Heller SL, Holbrook AI, Lee SJ, Lewin AA, Expert Panel on Breast I (2017). ACR appropriateness criteria((R)) monitoring response to neoadjuvant systemic therapy for breast cancer. J Am Coll Radiol.

[10] Ecanow JS, Abe H, Newstead GM, Ecanow DB, Jeske JM (2013). Axillary staging of breast cancer: what the radiologist should know. Radiographics.

[11] Bröstcancer -Nationellt vårdprogram 2020, https://kunskapsbanken.cancercentrum.se/globalassets/cancerdiagnoser/brost/vardprogram/nationellt-vardprogram-brostcancer.pdf. Accessed 30 Sept 2020

[12] Liedtke C, Jackisch C, Thill M, Thomssen C, Muller V, Janni W, Committee AGOB (2018). AGO recommendations for the diagnosis and treatment of patients with early breast cancer: update 2018. Breast Care (Basel).

[13] Cardoso F, Kyriakides S, Ohno S, Penault-Llorca F, Poortmans P, Rubio IT, Zackrisson S, Senkus E (2019). Early breast cancer: ESMO Clinical Practice Guidelines for diagnosis, treatment and follow-updagger. Ann Oncol.

[14] Alvarez S, Anorbe E, Alcorta P, Lopez F, Alonso I, Cortes J (2006). Role of sonography in the diagnosis of axillary lymph node metastases in breast cancer: a systematic review. Am J Roentgenol.

[15] Marino MA, Avendano D, Zapata P, Riedl CC, Pinker K (2020). Lymph node imaging in patients with primary breast cancer: concurrent diagnostic tools. Oncologist.

[16] Schwentner L, Helms G, Nekljudova V, Ataseven B, Bauerfeind I, Ditsch N, Fehm T, Fleige B, Hauschild M, Heil J (2017). Using ultrasound and palpation for predicting axillary lymph node status following neoadjuvant chemotherapy—results from the multi-center SENTINA trial. Breast.

[17] Kim HS, Shin MS, Kim CJ, Yoo SH, Yoo TK, Eom YH, Chae BJ, Song BJ (2017). Improved model for predicting axillary response to neoadjuvant chemotherapy in patients with clinically node-positive breast cancer. J Breast Cancer.

[18] Ha SM, Cha JH, Kim HH, Shin HJ, Chae EY, Choi WJ (2017). Diagnostic performance of breast ultrasonography and MRI in the prediction of lymph node status after neoadjuvant chemotherapy for breast cancer. Acta Radiol.

[19] Boileau JF, Poirier B, Basik M, Holloway CM, Gaboury L, Sideris L, Meterissian S, Arnaout A, Brackstone M, McCready DR (2015). Sentinel node biopsy after neoadjuvant chemotherapy in biopsy-proven node-positive breast cancer: the SN FNAC study. J Clin Oncol.

[20] Morrow E, Lannigan A, Doughty J, Litherland J, Mansell J, Stallard S, Mallon E, Romics L (2018). Population-based study of the sensitivity of axillary ultrasound imaging in the preoperative staging of node-positive invasive lobular carcinoma of the breast. Br J Surg.

[21] Upadhyaya VS, Lim GH, Chan EYK, Fook-Chong SMC, Leong LCH (2020). Evaluating the preoperative breast cancer characteristics affecting the accuracy of axillary ultrasound staging. Breast J.

[22] Dihge L, Grabau DA, Rasmussen RW, Bendahl PO, Ryden L (2016). The accuracy of preoperative axillary nodal staging in primary breast cancer by ultrasound is modified by nodal metastatic load and tumor biology. Acta Oncol.

[23] Saal LH, Vallon-Christersson J, Hakkinen J, Hegardt C, Grabau D, Winter C, Brueffer C, Tang MH, Reutersward C, Schulz R (2015). The Sweden Cancerome Analysis Network - Breast (SCAN-B) Initiative: a large-scale multicenter infrastructure towards implementation of breast cancer genomic analyses in the clinical routine. Genome Med.

[24] Skarping I, Fornvik D, Heide-Jorgensen U, Sartor H, Hall P, Zackrisson S, Borgquist S (2020). Mammographic density changes during neoadjuvant breast cancer treatment: NeoDense, a prospective study in Sweden. Breast.

[25] Skarping I, Fornvik D, Heide-Jorgensen U, Ryden L, Zackrisson S, Borgquist S (2020). Neoadjuvant breast cancer treatment response; tumor size evaluation through different conventional imaging modalities in the NeoDense study. Acta Oncol.

[26] Bossuyt V, Provenzano E, Symmans WF, Boughey JC, Coles C, Curigliano G, Dixon JM, Esserman LJ, Fastner G, Kuehn T (2015). Recommendations for standardized pathological characterization of residual disease for neoadjuvant clinical trials of breast cancer by the BIG-NABCG collaboration. Ann Oncol.

[27] Krishnamurthy S, Sneige N, Bedi DG, Edieken BS, Fornage BD, Kuerer HM, Singletary SE, Hunt KK (2002). Role of ultrasound-guided fine-needle aspiration of indeterminate and suspicious axillary lymph nodes in the initial staging of breast carcinoma. Cancer.

[28] Dialani V, James DF, Slanetz PJ (2015). A practical approach to imaging the axilla. Insights Imaging.

[29] Park SH, Jeong YM, Cho SH, Jung HK, Kim SJ, Ryu HS (2014). Imaging findings of variable axillary mass and axillary lymphadenopathy. Ultrasound Med Biol.

[30] Sickles E, D’Orsi CJ, Bassett LW (2013). ACR BI-RADS® mammography. ACR BI-RADS® atlas, breast imaging reporting and data system.

[31] Highnam R, Brady SM, Yaffe MJ, Karssemeijer N, Harvey J (2010). Robust breast composition measurement—VolparaTM.

[32] Vila J, Mittendorf EA, Farante G, Bassett RL, Veronesi P, Galimberti V, Peradze N, Stauder MC, Chavez-MacGregor M, Litton JF (2016). Nomograms for predicting axillary response to neoadjuvant chemotherapy in clinically node-positive patients with breast cancer. Ann Surg Oncol.

[33] Schipper RJ, Moossdorff M, Nelemans PJ, Nieuwenhuijzen GA, de Vries B, Strobbe LJ, Roumen RM, van den Berkmortel F, Tjan-Heijnen VC, Beets-Tan RG (2014). A model to predict pathologic complete response of axillary lymph nodes to neoadjuvant chemo(immuno)therapy in patients with clinically node-positive breast cancer. Clin Breast Cancer.

[34] Murphy BL, Hoskin TL, Heins CDN, Habermann EB, Boughey JC (2017). Preoperative prediction of node-negative disease after neoadjuvant chemotherapy in patients presenting with node-negative or node-positive breast cancer. Ann Surg Oncol.

[35] Jin X, Jiang YZ, Chen S, Shao ZM, Di GH (2016). A nomogram for predicting the pathological response of axillary lymph node metastasis in breast cancer patients. Sci Rep.

[36] Kantor O, Sipsy LM, Yao K, James TA (2018). A predictive model for axillary node pathologic complete response after neoadjuvant chemotherapy for breast cancer. Ann Surg Oncol.

[37] Kim JY, Park HS, Kim S, Ryu J, Park S, Kim SI (2015). Prognostic nomogram for prediction of axillary pathologic complete response after neoadjuvant chemotherapy in cytologically proven node-positive breast cancer. Medicine (Baltimore).

[38] Ouldamer L, Chas M, Arbion F, Body G, Cirier J, Ballester M, Bendifallah S, Darai E (2018). Risk scoring system for predicting axillary response after neoadjuvant chemotherapy in initially node-positive women with breast cancer. Surg Oncol.

[39] Choi HY, Park M, Seo M, Song E, Shin SY, Sohn YM (2017). Preoperative axillary lymph node evaluation in breast cancer: current issues and literature review. Ultrasound Q.

[40] Le-Petross HT, McCall LM, Hunt KK, Mittendorf EA, Ahrendt GM, Wilke LG, Ballman KV, Boughey JC (2018). Axillary ultrasound identifies residual nodal disease after chemotherapy: results from the American College of Surgeons Oncology Group Z1071 Trial (Alliance). Am J Roentgenol.

[41] Ganeshalingam S, Koh DM (2009). Nodal staging. Cancer Imaging.

[42] Weber JJ, Jochelson MS, Eaton A, Zabor EC, Barrio AV, Gemignani ML, Pilewskie M, Van Zee KJ, Morrow M, El-Tamer M (2017). MRI and prediction of pathologic complete response in the breast and axilla after neoadjuvant chemotherapy for breast cancer. J Am Coll Surg.

[43] Hieken TJ, Boughey JC, Jones KN, Shah SS, Glazebrook KN (2013). Imaging response and residual metastatic axillary lymph node disease after neoadjuvant chemotherapy for primary breast cancer. Ann Surg Oncol.

[44] Giovagnorio F, Drudi FM, Fanelli G, Flecca D, Francioso A (2005). Fatty changes as a misleading factor in the evaluation with ultrasound of superficial lymph nodes. Ultrasound Med Biol.

[45] Shah AR, Glazebrook KN, Boughey JC, Hoskin TL, Shah SS, Bergquist JR, Dupont SC, Hieken TJ (2014). Does BMI affect the accuracy of preoperative axillary ultrasound in breast cancer patients?. Ann Surg Oncol.

[46] Kim R, Chang JM, Lee HB, Lee SH, Kim SY, Kim ES, Cho N, Moon WK (2019). Predicting axillary response to neoadjuvant chemotherapy: breast MRI and US in patients with node-positive breast cancer. Radiology.

[47] Boughey JC, McCall LM, Ballman KV, Mittendorf EA, Ahrendt GM, Wilke LG, Taback B, Leitch AM, Flippo-Morton T, Hunt KK (2014). Tumor biology correlates with rates of breast-conserving surgery and pathologic complete response after neoadjuvant chemotherapy for breast cancer: findings from the ACOSOG Z1071 (Alliance) prospective multicenter clinical trial. Ann Surg.

[48] Huo CW, Chew GL, Britt KL, Ingman WV, Henderson MA, Hopper JL, Thompson EW (2014). Mammographic density-a review on the current understanding of its association with breast cancer. Breast Cancer Res Treat.

[49] Sherratt MJ, McConnell JC, Streuli CH (2016). Raised mammographic density: causative mechanisms and biological consequences. Breast Cancer Res.

[50] Bae MS, Moon HG, Han W, Noh DY, Ryu HS, Park IA, Chang JM, Cho N, Moon WK (2016). Early stage triple-negative breast cancer: imaging and clinical-pathologic factors associated with recurrence. Radiology.

[51] Skarping I, Fornvik D, Sartor H, Heide-Jorgensen U, Zackrisson S, Borgquist S (2019). Mammographic density is a potential predictive marker of pathological response after neoadjuvant chemotherapy in breast cancer. BMC Cancer.

[52] Elsamany S, Alzahrani A, Abozeed WN, Rasmy A, Farooq MU, Elbiomy MA, Rawah E, Alsaleh K, Abdel-Aziz NM (2015). Mammographic breast density: predictive value for pathological response to neoadjuvant chemotherapy in breast cancer patients. Breast.

[53] Bertrand KA, Tamimi RM, Scott CG, Jensen MR, Pankratz V, Visscher D, Norman A, Couch F, Shepherd J, Fan B (2013). Mammographic density and risk of breast cancer by age and tumor characteristics. Breast Cancer Res.

[54] van la Parra RF, Peer PG, Ernst MF, Bosscha K (2011). Meta-analysis of predictive factors for non-sentinel lymph node metastases in breast cancer patients with a positive SLN. Eur J Surg Oncol.

[55] Majid S, Ryden L, Manjer J (2019). Determinants for non-sentinel node metastases in primary invasive breast cancer: a population-based cohort study of 602 consecutive patients with sentinel node metastases. BMC Cancer.

[56] Zhang L, Liu C, Wang W, Xu X, Chen B (2012). Is optimal timing of sentinel lymph node biopsy before neoadjuvant chemotherapy in patients with breast cancer? A literature review. Surg Oncol.

[57] Zetterlund L, Celebioglu F, Axelsson R, de Boniface J, Frisell J (2017). Swedish prospective multicenter trial on the accuracy and clinical relevance of sentinel lymph node biopsy before neoadjuvant systemic therapy in breast cancer. Breast Cancer Res Treat.

[58] Ashikaga T, Krag DN, Land SR, Julian TB, Anderson SJ, Brown AM, Skelly JM, Harlow SP, Weaver DL, Mamounas EP (2010). Morbidity results from the NSABP B-32 trial comparing sentinel lymph node dissection versus axillary dissection. J Surg Oncol.

[59] Krag DN, Anderson SJ, Julian TB, Brown AM, Harlow SP, Costantino JP, Ashikaga T, Weaver DL, Mamounas EP, Jalovec LM (2010). Sentinel-lymph-node resection compared with conventional axillary-lymph-node dissection in clinically node-negative patients with breast cancer: overall survival findings from the NSABP B-32 randomised phase 3 trial. Lancet Oncol.

[60] Eisenhauer EA, Therasse P, Bogaerts J, Schwartz LH, Sargent D, Ford R, Dancey J, Arbuck S, Gwyther S, Mooney M (2009). New response evaluation criteria in solid tumours: revised RECIST guideline (version 1.1). Eur J Cancer.

[61] Pyo JS, Jung J, Lee SG, Kim NY, Kang DW (2020). Diagnostic accuracy of fine-needle aspiration cytology and core-needle biopsy in the assessment of the axillary lymph nodes in breast cancer—a meta-analysis. Diagnostics (Basel).

