# Supplementary material for: Predicting pathological axillary lymph node status with ultrasound following neoadjuvant therapy for breast cancer
Source: Breast Cancer Res Treat. 2021 Jun 12;189(1):131–44. doi: 10.1007/s10549-021-06283-8 (PMC8302508; doi:10.1007/s10549-021-06283-8)
Supplement: Supplementary file 1 — Supplementary file1 Study time line and treatment algorithm (PPTX 4476 kb) [file 10549_2021_6283_MOESM1_ESM.pptx]

## Slide 1
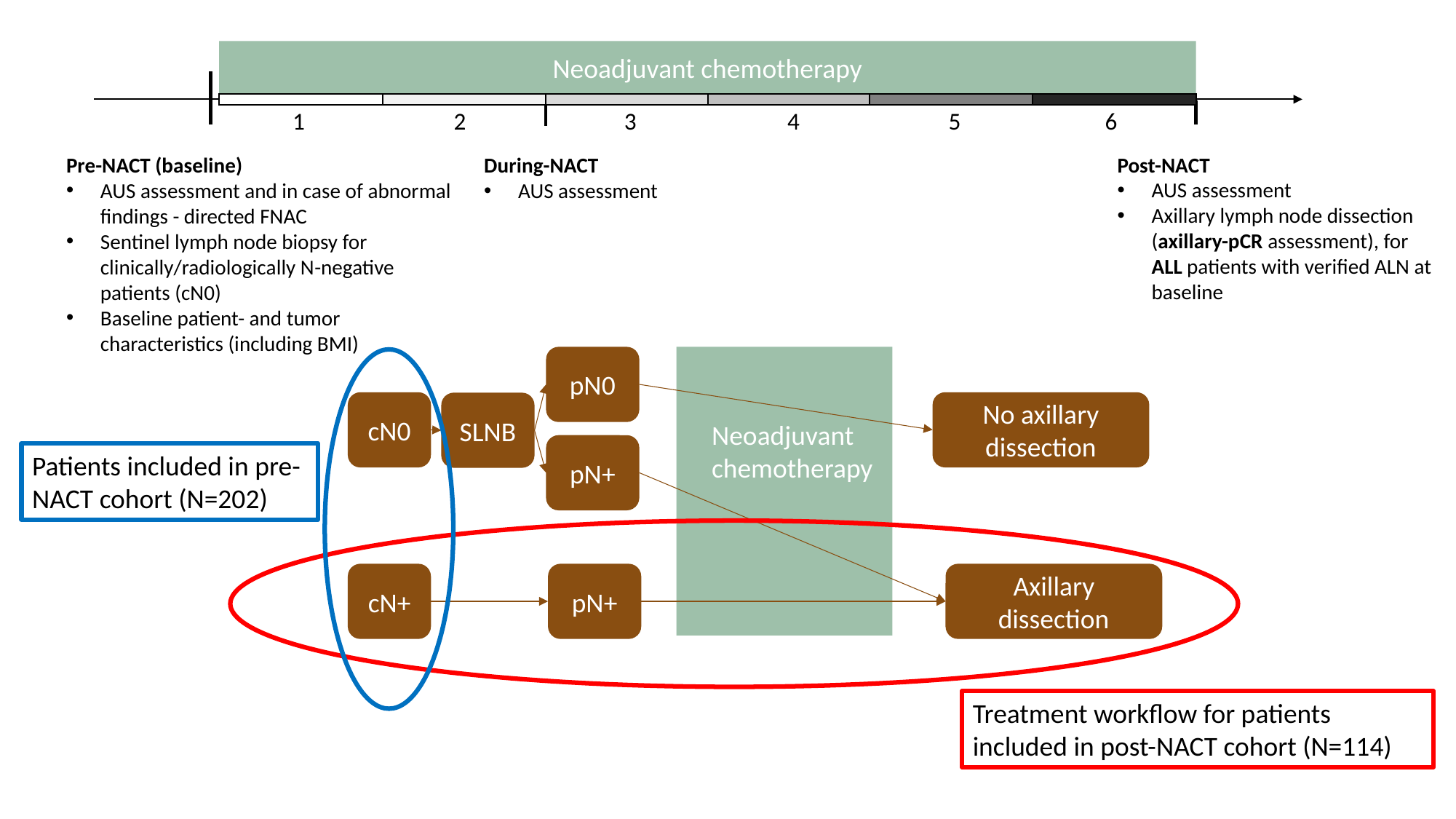

Neoadjuvant chemotherapy
1
3
5
2
6
4
Post-NACT
AUS assessment
Axillary lymph node dissection (axillary-pCR assessment), for ALL patients with verified ALN at baseline
Pre-NACT (baseline)
AUS assessment and in case of abnormal findings - directed FNAC
Sentinel lymph node biopsy for clinically/radiologically N-negative patients (cN0)
Baseline patient- and tumor characteristics (including BMI)
During-NACT
AUS assessment
pN0
No axillary dissection
cN0
SLNB
Neoadjuvant
chemotherapy
pN+
Patients included in pre-NACT cohort (N=202)
cN+
pN+
Axillary dissection
Treatment workflow for patients included in post-NACT cohort (N=114)
