# Supplementary material for: Predicting pathological axillary lymph node status with ultrasound following neoadjuvant therapy for breast cancer
Source: Breast Cancer Res Treat. 2021 Jun 12;189(1):131–44. doi: 10.1007/s10549-021-06283-8 (PMC8302508; doi:10.1007/s10549-021-06283-8)
Supplement: Supplementary file 2 — Supplementary file2 (DOCX 15 kb) [file 10549_2021_6283_MOESM2_ESM.docx]

Supplementary Material 2. Details of the ultrasound machines used in the study.

|  | Start – Feb 2015 | Feb 2015 – Sep 2016 | Sep 2016 – Feb 2019 | Feb 2019 – end |
| --- | --- | --- | --- | --- |
| Site 1 | Siemens Sequoia 512 | Toshiba Aplio TUS-400 | GE E9 Logiq | GE E10 Logiq |
|  | Siemens S-2000 |  |  |  |
|  | The whole study time |  |  |  |
| Site 2 | Aloka prosound F75 |  |  |  |
|  | GE Logic E 10 |  |  |  |
