# Supplementary material for: Predicting pathological axillary lymph node status with ultrasound following neoadjuvant therapy for breast cancer
Source: Breast Cancer Res Treat. 2021 Jun 12;189(1):131–44. doi: 10.1007/s10549-021-06283-8 (PMC8302508; doi:10.1007/s10549-021-06283-8)
Supplement: Supplementary file 3 — Supplementary file3 (DOCX 30 kb) [file 10549_2021_6283_MOESM3_ESM.docx]

Supplementary Material 3. Post-NACT cohort: Simple and multivariable logistic regression analysis of imaging features of the breast and ALN pre-, during and post-NACT as predictors of **axillary pCR** following NACT.

|  | Variables | Simple logistic regression | | | Multivariable* logistic regression | | |
| --- | --- | --- | --- | --- | --- | --- | --- |
|  |  | OR (95% CI) | *p* value | N | OR (95% CI) | *p* value | N |
| Imaging characterisics | |  |  |  |  |  |  |
| Pre-NACT | US tumor size (mm) | 0.97 (0.95-1.01) | 0.21 | 112 | 0.97 (0.93-1.01) | 0.11 | 111 |
|  | Number of abnormal ALN by AUS | 1.19 (0.78-1.82) | 0.41 | 114 | 0.90 (0.53-1.53) | 0.70 | 113 |
|  | Longest abnormal ALN diamater | 1.01 (0.96-1.06) | 0.81 | 102 | 0.96 (0.90-1.02) | 0.19 | 101 |
|  | Ratio of long/short ALN diameter | 0.64 (0.25-1.60) | 0.34 | 79 | 0.94 (0.26-3.48) | 0.93 | 79 |
| - mammographic density | -Bi-RADS (ref A) | (ref) |  | 6 | (ref) |  | 6 |
|  | - B | 0.87 (0.14-5.34) | 0.88 | 43 | 1.56 (0.15-16.67) | 0.71 | 43 |
|  | - C | 0.91 (0.15-5.52) | 0.92 | 48 | 1.18 (0.11-12.93) | 0.89 | 47 |
|  | - D | 0.62 (0.08-4.70) | 0.64 | 17 | 0.83 (0.05-14.31) | 0.90 | 17 |
|  | -BI-RADS dichotomized (ref A/B ”non-dense”) | (ref) |  | 49 | (ref) |  | 49 |
|  | - C/D (”dense”) | 0.94 (0.42-2.10) | 0.87 | 65 | 0.73 (0.26-2.10) | 0.56 | 64 |
|  | -VBD% | 0.99 (0.95-1.06) | 0.98 | 110 | 0.94 (0.85-1.03) | 0.20 | 109 |
| During NACT | US tumor size (mm) | 0.96 (0.92-1.00) | 0.05 | 109 | 0.99 (0.94-1.03) | 0.54 | 108 |
|  | Tumor response (decrease ≥30%, yes/no) | 2.60 (1.11-6.07) | 0.03 | 107 | 1.66 (0.59-4.63) | 0.34 | 106 |
|  | Number of abnormal ALN by AUS | 0.81 (0.55-1.19) | 0.28 | 113 | 0.46 (0.25-0.83) | 0.01 | 112 |
|  | Longest abnormal ALN diameter | 1.01 (0.94-1.07) | 0.88 | 75 | 0.98 (0.91-1.06) | 0.60 | 74 |
|  | Ratio of long/short ALN diameter | 1.75 (0.88-3.49) | 0.11 | 57 | 5.77 (1.67-19.92) | <0.01 | 56 |
| - mammographic density | -Bi-RADS (ref A) | (ref) |  | 12 | (ref) |  | 12 |
|  | - B | 1.80 (0.33-9.84) | 0.50 | 34 | 1.26 (0.14-11.63) | 0.84 | 34 |
|  | - C | 3.23 (0.64-16.28) | 0.16 | 51 | 3.83 (0.40-36.26) | 0.24 | 50 |
|  | - D | 1.50 (0.21-11.00) | 0.69 | 13 | 1.36 (0.08-22.08) | 0.83 | 13 |
|  | -BI-RADS dichotomized (ref A/B ”non-dense”) | (ref) |  | 46 | (ref) |  | 46 |
|  | - C/D (”dense”) | 1.79 (0.76-4.17) | 0.18 | 64 | 2.70 (0.81-9.04) | 0.11 | 63 |
|  | -VBD% | 1.00 (0.94-1.06) | 0.94 | 109 | 0.95 (0.86-1.05) | 0.30 | 108 |
|  | Relative change in VBD%** | 1.11 (0.95-1.30) | 0.18 | 105 | 1.10 (0.91-1.33) | 0.32 | 104 |
| Post-NACT | US tumor size (mm) | 0.95 (0.90-1.00) | 0.04 | 111 | 0.99 (0.94-1.04) | 0.59 | 110 |
|  | Tumor response (decrease ≥30% (compared to baseline) | 2.46 (0.51-11.80) | 0.26 | 110 | 2.02 (0.35-11.58) | 0.43 | 109 |
|  | Tumor response (decrease ≥70% compared to baseline) | 2.89 (1.23-6.76) | 0.02 | 110 | 1.28 (0.45-3.66) | 0.65 | 109 |
|  | Number of abnormal ALN by AUS | 0.66 (0.39-1.13) | 0.13 | 113 | 0.58 (0.30-1.10) | 0.10 | 112 |
|  | Longest abnormal ALN diameter | 1.20 (1.00-1.44) | 0.05 | 40 | 1.15 (0.97-1.37) | 0.12 | 40 |
|  | Ratio of long/short ALN diameter | 2.49 (0.84-7.38) | 0.10 | 30 | 2.53 (0.57-11.11) | 0.22 | 30 |
| - mammographic density | -Bi-RADS (ref A) | (ref) |  | 7 | (ref) |  | 7 |
|  | - B | 1.46 (0.15-13.85) | 0.75 | 41 | 1.84 (0.10-33.21) | 0.68 | 41 |
|  | - C | 4.00 (0.44-36.08) | 0.22 | 45 | 9.51 (0.47-192.63) | 0.14 | 44 |
|  | - D | 3.75 (0.34-41.08) | 0.28 | 13 | 23.47 (0.68-807.23) | 0.08 | 13 |
|  | -BI-RADS dichotomized (ref A/B ”non-dense”) | (ref) |  | 48 | (ref) |  | 48 |
|  | - C/D (”dense”) | 2.85 (1.16-6.97) | 0.02 | 58 | 5.65 (1.49-21.39) | 0.01 | 57 |
|  | -VBD% | 1.01 (0.94-1.08) | 0.89 | 108 | 0.94 (0.84-1.05) | 0.25 | 107 |
|  | Relative change in VBD%*** | 1.01 (0.91-1.13) | 0.86 | 104 | 1.05 (0.90-1.22) | 0.53 | 103 |

* Included variabels: age+ BMI + menopausal status + ER + HER2 + Ki67

**$\frac{(VBD\% at T1)- (VBD\% at T0)}{(VBD\% at T0)}$, OR correspond to a 5% change

***$\frac{(VBD\% at T2)- (VBD\% at T0)}{(VBD\% at T0)}$, OR correspond to a 5% change

Abbreviations: neoadjuvant chemotherapy (NACT), axillary lymph nodes (ALN), pathological complete response (pCR), odds ratio (OR), confidence interval (CI), ultrasound (US), axillary ultrasound (AUS), Breast Imaging-Reporting and Data System (BI-RADS), volumetric breast density percentage (VBD%), body mass index (BMI), estrogen receptor (ER), human epidermal growth factor receptor 2 (HER2)
